# Yaws in Africa: Past, Present and Future

**DOI:** 10.3390/diseases13010014

**Published:** 2025-01-14

**Authors:** Ezekiel K. Vicar, Shirley V. Simpson, Gloria I. Mensah, Kennedy K. Addo, Eric S. Donkor

**Affiliations:** 1 Department of Clinical Microbiology, University for Development Studies, Tamale P.O. Box TL 1350, Ghana; 2 Department of Medical Microbiology, University of Ghana Medical School, Accra P.O. Box GP 4236, Ghana; 3 Department of Bacteriology, Noguchi Memorial Institute for Medical Research, University of Ghana, Legon, Accra P.O. Box LG 581, Ghana; ssimpson@noguchi.ug.edu.gh (S.V.S.);

**Keywords:** yaws, *Treponema pallidum* subsp. *pertenue*, treponematosis, eradication, neglected tropical diseases

## Abstract

**Background:** Yaws is an infectious, neglected tropical disease that affects the skin of many children and adolescents who live in poor, rural, low-income communities in humid, tropical areas of Africa, Southeast Asia, and the Pacific Islands. Yaws is currently endemic in at least 15 countries, but adequate surveillance data are lacking. In line with the WHO’s effort to improve early detection, diagnosis, and proper management leading to the eventual eradication of yaws, this article reviews the existing literature on yaws in Africa to highlight the epidemiological pattern, genetic variability, diagnosis modalities, treatment, and control strategies, the challenges and prospects for yaws eradication. **Methods**: We searched PubMed and Scopus databases to identify published data in line with the review objectives. **Results:** One hundred and eighty-eight peer-reviewed articles were identified by PubMed and Scopus, out of which thirty were eligible. The studies covered 11 African countries, with the reported prevalence ranging from 0.50% to 43.0%. **Conclusions**: There is a great prospect for eradication if countries capitalize on the availability of simple, inexpensive, and well-tolerated oral treatment that has proven effective, validated point-of-care diagnostic tests and new molecular tests. Countries should embark on integrated disease control efforts to increase sustainability and improve the quality of life for people living with this NTD in poor communities.

## 1. Introduction

Yaws is an infectious neglected tropical disease (NTD) caused by a corkscrew-shaped Gram-negative bacterium *Treponema pallidum* subspecies *pertenue* (TPE), a close relative of other *Treponema* bacteria, including those that cause syphilis. This bacterium was discovered by a physician, Castellani Aldo, in 1905 after examining scrapings from yaws lesions from patients in Sri Lanka [1] and later confirming it from lesions of a patient with yaws from Africa [2]. Yaws initially manifest as a wart-like skin tumor that later becomes lesions. The bacterium from a lesion can spread among people through direct skin contact. Many lesions might heal spontaneously without treatment due to the host’s immunity. However, the bacterium can sometimes lie dormant in the host for years. In about 10% of untreated and immunocompromised patients, reemergence can cause painful inflammation and destruction of bone and surrounding tissue, leading to disfigurement and eventual disability [3,4]. The nature of this chronic, disfiguring, and debilitating childhood infectious neglected tropical disease could be disturbing as it has the potential to curtail the education of affected children and limit social inclusiveness [4].

Yaws is considered endemic in humid, tropical areas of Africa, Southeast Asia, and the Pacific Islands and affects the skin of children under 15 years who live in poor, rural, and low-income communities [4,5]. The WHO and UNICEF’s first significant yaws eradication effort occurred from 1952 to 1964. The strategy was a mass treatment campaign in 46 countries [3,6,7], where more than 50 million cases out of the 300 million people screened received treatment with benzathine penicillin [6,8]. The campaign caused about a 95% reduction in the global burden of yaws [6]. By the end of the 1970s, yaws had re-emerged in Africa due to the integration of yaws eradication activities into national public health systems, lack of focus on yaws, failed surveillance, and low commitment towards yaws eradication [9,10]. Lack of governmental commitment and resources to reach endemic communities were also seen as the major contributor to failed surveillance and reduced commitment in most countries [10]. Also, the intramuscular benzathine penicillin injection was not well tolerated by most children who are the burden bearers of yaws. In 2012, results from randomized non-inferiority clinical trials conducted in Ghana and Papua New Guinea showed that a single oral dose of azithromycin (30 mg/kg) was effective and non-inferior to intramuscular benzathine penicillin and was well tolerated [11,12]. Based on these findings, azithromycin became the WHO-preferred treatment for yaws [13]. The WHO developed the “Morges Strategy”, which was based on total community treatment (TCT) as well as case finding and targeted treatment with azithromycin to eradicate yaws [13]. Subsequently, in 2014, Ayove, et al. [14] showed that the sensitivity of a point-of-care immunoassay, the Dual Path Platform (DPP) syphilis assay, was accurate enough to detect antibodies to treponemal and non-treponemal antigens in patients with yaws. This immunoassay is used to guide antibiotics usage for yaws eradication.

Despite the success, challenges such as a lack of resources to reach and treat people in remote areas and to monitor the disease [15] and several uncertainties related to the biology and epidemiology of yaws [4,7,16] can cause a rollback. In line with the WHO’s effort to improve early detection, diagnosis, and proper management leading to the eventual eradication of yaws by 2030, this article reviews the existing literature on yaws in Africa, to highlight the epidemiological pattern, genetic variability, diagnosis modalities, treatment, and control strategies, the challenges and prospects for yaws eradication.

## 2. Materials and Methods

Peer-reviewed articles focused on yaws epidemiology (prevalence, incidence, and regional distribution), genetic variability, diagnosis modalities, and treatment and control strategies were reviewed based on the objectives of this review. Article hunts were categorized into (1) a search plan, (2) selection of relevant articles, (3) charting the article selection process, and (4) summarizing the major findings of selected articles in a tabular form. This study was not registered on any platform.

### 2.1. Search Plan

Well-established databases, including PubMed and Scopus, were explored for relevant articles using constructed Boolean search strings for each objective. “Yaws” OR “treponematosis” AND “prevalence” OR “eradication programme” OR “antibiotic resistance” OR “diagnostic methods” OR “genetics”. Specific search strings were formulated to capture articles relevant to each objective. Additional filters were applied to eliminate review articles. Titles and abstracts of articles were reviewed to assess their relevance. Full-text articles for relevant studies were retrieved and managed using EndNote Reference Manager. Additionally, references to relevant articles were reviewed to identify more pertinent literature. The scope of article selection captures published peer-reviewed articles, experimental studies, clinical trials, and preclinical studies. Studies related to yaws in Africa were captured using guidelines for the Preferred Reporting Items for Systematic Reviews and Meta-Analysis (PRISMA).

### 2.2. Selection of Relevant Articles

**Figure 1:**
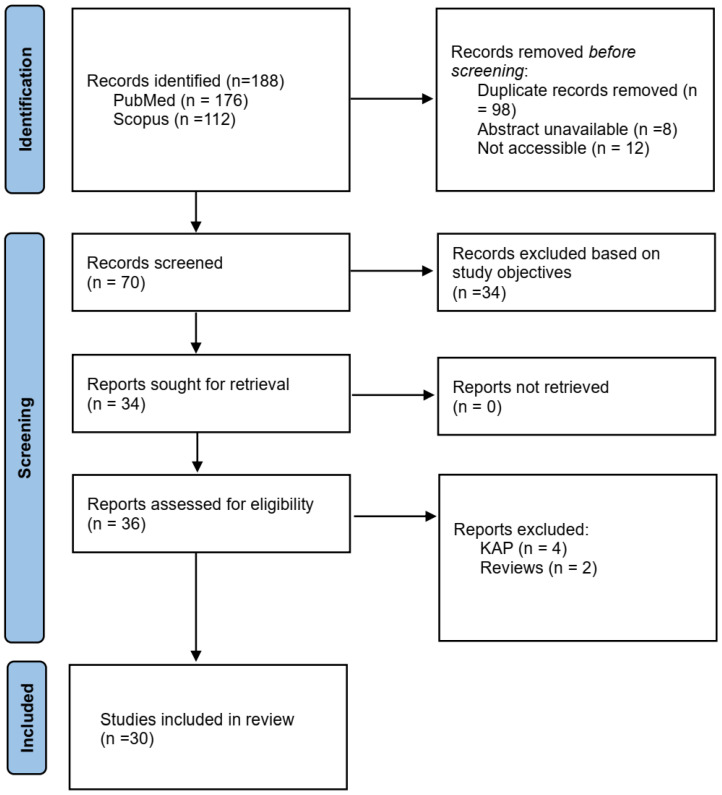
PRISMA flow diagram for identification, screening, and evaluation of articles in the study.

Articles included were those published between 1990 and 2024. First, two individuals screened the titles and abstracts, and articles that met the inclusion criteria were considered for review. A third reviewer resolved discrepancies. The inclusion criteria were full peer-reviewed articles written in English, focusing on yaws in Africa. The excluded articles were those written in languages other than English or those without full articles. The PRISMA flow chart in Figure 1 shows the procedural selection of relevant articles.

## 3. Results

### 3.1. Search Results

The following information was extracted from the articles which met the inclusion criteria: author/authors’ name, year of publication, study type, study focus, study location, and significant study findings. Findings were organized according to key themes, and gaps in the literature were identified for future research that addressed potential limitations of the existing literature. The results of this review are presented in six subheadings: (1) an overview of selected studies; (2) prevalence, incidence, and distribution of yaws in Africa; (3) diagnosis of yaws; (4) genetic variability of *T. pallidum* subsp. *pertenue*; (5) treatment and antibiotic resistance; and (6) control strategies of yaws in Africa and associated challenges.

### 3.2. Overview of Selected Studies

With our search criteria, 188 peer-reviewed articles were identified from PubMed and Scopus. A summary of a detailed review of 30 eligible articles is presented in Table 1. The studies covered 11 countries in Africa, including Benin [17], Burkina Faso [17], Cameroon, Central Africa Republic (CAR) [18,19,20], Congo [21,22], Côte d’Ivoire [23], Democratic Republic of Congo (DRC) [22], Ghana [10,24,25], Liberia [26], Nigeria [27,28], and Togo [29]. Eight studies were community-based [17,19,21,22,23,24,26,28], while three were school-based [10,18,21]. Two studies combined community and school-based [27,29] approaches, and one was hospital-based [25].

### 3.3. Objectives-Based Analysis of the Results

#### 3.3.1. Prevalence, Incidence, and Distribution of Yaws in Africa

The results of the thirteen (13) studies that were found eligible showed the prevalence of yaws ranging between 0.50% and 43.0% [10,17,18,19,21,22,23,24,25,26,27,28,29], except for one study conducted in Nigeria that recorded no yaws cases [27]. In Ghana, studies into the prevalence of yaws were conducted in the following regions: Eastern, Central, Volta, Western, Ashanti, and Brong-Ahafo [24,25]. In Cote d’Ivoire, studies covered the entire country [23] (Table 1).

Five studies [26,30,31,32,33] reported the incidence of yaws in studies conducted in health facilities (Table 2).

#### 3.3.2. Diagnosis of Yaws

Diagnosing yaws is performed clinically, serologically, or using molecular methods. In two studies reviewed, patients were diagnosed solely on clinical symptoms [10,28]. In nine studies, suspected cases with clinical signs of yaws were further tested serologically using VDRL, TPHA, RPR, and DPP tests [17,18,19,21,22,23,25,26,27]. Two studies conducted in Ghana used molecular methods [24,25].

#### 3.3.3. Genetic Variability of *T. pallidum* Subsp. *pertenue*

One study from Liberia used whole genome sequencing to characterize *T. pallidium* subsp. *pertenue* [26] identified extremely closely related monophyletic clades different from those found in the TPE genome from humans and nonhuman primates [34]. Marks, et al. [35] used the new generation sequencing (NGS) method in their phylogenetic analysis to demonstrate that *T. pallidum* subsp. *pertenue* strains from the Solomon Islands and Ghana form a discrete lineage that can be further subdivided into two distinct clades, both of which are distinct from all previously sequenced *T. pallidum* subsp. *pertenue* samples. Again, three recombinant genomic regions were identified. Region 1 included the TPESAMD_0134 gene predicted to encode a putative outer member protein. Region 2 was predicted to encode ten genes and two gene remnants, including tp0858. Region 3 encompassed the tprK gene, known in *T. pallidum* subsp. *pallidum* to undergo antigenic variation via segmental gene conversion [35]. In another study conducted in Ghana and Vanuatu, nine strain types (3q12/ah, 4r12/ah, 4q10/j, 4q11/ah, 4q12/ah, 4q12/v, 4q13/ah, 6q10/aj, and 9q10/ai) were identified in clinical samples from Ghana [34] (Table 3).

#### 3.3.4. Treatment of Yaws and Antibiotic Resistance in Africa

In terms of treatment, a randomized non-inferiority trial conducted in Ghana compared the efficacy of a single oral dose of azithromycin as an alternative to intramuscular benzathine penicillin for the treatment of yaws and concluded that azithromycin could be used for mass drug administration in resource-limited settings [12]. Following this trial, Abdulai, et al. [36] piloted a community-based mass treatment with azithromycin to eliminate yaws in Ghana, reducing the prevalence of serologically confirmed active yaws from 5.7% to 0.6%. Subsequently, in a randomized non-inferiority trial in Ghana and Papua New Guinea, Marks, et al. [37] conducted a study comparing the efficacy of low-dose versus standard-dose azithromycin for patients with yaws. They found that low-dose azithromycin did not meet the prespecified non-inferiority margin compared with standard-dose azithromycin in achieving clinical and serological cure in PCR-confirmed active yaws that 20 mg/kg of azithromycin is probably effective against yaws. From the data retrieved, only twelve studies stated the treatment regimen used after confirming suspected yaws cases. Six studies [17,18,19,20,25,38] used azithromycin for all confirmed cases, while six other studies used benzathine penicillin [10,22,23,25,28,39].

In Liberia, a report from in silico analysis predicted no resistance to the macrolide based on the analysis of the resistance loci A2058G and A2059G in the 23S ribosomal regions previously reported for syphilis or yaws [26]. So far, no data in Africa show any evidence of macrolide resistance in *T. pallidum* subsp. *pertenue.*

#### 3.3.5. Control Strategies of Yaws in Africa

Since the publication of the “Morges strategy” for the global eradication of yaws, a pilot implementation was conducted in the Abamkrom sub-district of Ghana, where 89% of the 16,287 people received a single oral dose of azithromycin [13,36]. Recently, the first significant implementation has been conducted in the Central Africa region, specifically in the Congo Basin, which 1,530,014 people inhabit [40]. The adoption of the strategy across Africa, especially in known endemic countries, remains limited [41].

**Table 1:** Prevalence and distribution of yaws in Africa.

Country	Reference	Year of Study	Location	Study Population *	Cases (Sample Size)	Diagnostic Method	Prevalence, %	Active/Latent
Burkina Faso	[17]	2018	Bagré and Kompienga	Community	28 (413)	RPR and TPHA	6.08	active
Cameroon	[38]	2023	Eastern region and Southern region	Community, school children,	(18/210)	RDT and DPP	8.6	Active
Central African Republic	[18]	2020	BaAka Pygmy	School children	494	Clinical	38.7	active
Central African Republic	[18]	2020	Bantu	School children	235	Clinical	43.00	active
Central African Republic	[19]	2020	Mbaïki	Community	41 (1967)	RPR and TPHA	2.08	active
Central African Republic	[39]	1990	Lobaye	School children	42 (213)	VDRL and TPHA	19.72	latent
Central African Republic	[39]	1990	Lobaye	School children	12 (213)	VDRL and TPHA	5.63	active
Côte d’Ivoire	[23]	2004	Adzopé	Community	11 (2182)	RPR	0.50	active
Côte d’Ivoire,	[38]	2023	Haaut sassandra, Béliér, Loh Djiboua, Agneby Tiassa	Community, school children,	(84/1232)	RDT and DPP	6.8%	active
Democratic Republic of the Congo	[22]	2005	Wasolo	Community	56 (1176)	RPR and TPHA	4.76	active
Ghana	[24]	2019	Oti region	community	10 (101)	PCR	10.85	active
Ghana	[25]	2021	Ashanti region	Hospital	19/110	DPP and PCR	17.3	active
Ghana	[10]	2008	Eastern Region	School children	4006 (208,413)	Clinical	1.92	active
Ghana	[38]	2023	Central region, Western region, and Eastern region	Community and school	(440/1643)	RDT and DPP	26.8%	active
Liberia	[26]	2018	Maryland County	Community	24 (1000)	Serological (DPP)	2.6	active
Nigeria	[27]	2021	Old Nsukka	Community and school	0 (7706)	RDT	0	active
Nigeria	[28]	1998	Garkida	Community	64 (1523)	Clinical	4.20	active
Republic of Congo	[21]	2012	Bétou, Ebyellé	Community	183 (6215)	RDT	2.94	active
Togo	[29]	2021	Ndjéi	Community and school	16 (1401)	Clinical	1.10	active

* Study population here denotes whether the study was conducted among members of a community or school children. VDRL = Venereal Disease Research Laboratory; TPHA = Treponema Pallidum Hemagglutination; RPR = Rapid Plasma region; RDT = Rapid Diagnostic Tests; DPP = Dual Path Platform; PCR = Polymerase chain reaction.

**Table 2:** Characteristics of studies on the incidence of Yaws.

Country	Reference	Year of Study	Location	Population	Method	Positive Cases (Number Screened)	Incidence
Côte d’Ivoire	[30]	2000	countrywide	adults and children	Clinical	9212 (15,882,758)	0.58
Côte d’Ivoire	[31]	2011	countrywide	adults and children	Clinical	3343 (22,594, 212)	0.15
Liberia	[26]	2018	countrywide	adults and children	PCR	3750 (3,787,000)	2.60
Nigeria	[32]	1999–2001	Countrywide	adults and children	Serology	0(2871)	0.00
Togo	[32]	1991	Nationwide	school children	Clinical	3750 (3787,000)	0.99

**Table 3:** Characteristics of genetic variability studies of yaws.

Country (Reference)	Year of Study	Study Objective	Major Outcome
Liberia [26]	2018	1. Used whole genome sequencing to characterize *Treponema pallidium* subsp. *pertenue* which identified an extremely closely related monophyletic clade different from those available in TPE genome form Humans and Nonhuman primates.	1. Liberia genomes form a monophyletic clade, genetically distant from publicly available genomes including three isolated from nonhuman primates in nearby Taï National Park (Côte d’Ivoire). 2. No Azithromycin resistance in the country. In silico analysis predicted no resistance based on the analysis of the resistance loci A2058G and A2059G in the 23S ribosomal regions previously reported for syphilis or yaws
Ghana and Solomon Island [35]	2013 and 2014	To employ next-generation sequencing to explore the reasons why *T. pallidum* subsp. *pertenue* was not detected using the 2015 CDC real-time PCR assay in samples from patients showing clinical and serological evidence of yaws, and to develop a modified assay capable of detecting these missed samples.	1. Phylogenetic analysis demonstrated that *T. pallidum* subsp. *pertenue* strains from the Solomon Islands form a discrete lineage that can be further subdivided into two distinct clades, both of which are distinct from all previously sequenced *T. pallidum* subsp. *pertenue* samples. 2. Identified three recombinant genomic regions. Region 1 included the TPESAMD_0134 gene predicted to encode a putative outer member protein.). Region 2 was predicted to encode 10 genes and 2 gene remnants, including tp0858. Region 3 encompassed the tprK gene, which is known in *T. pallidum* subsp. *pallidum* to undergo antigenic variation via segmental gene conversion
Ghana and Vanuatu [34]	2018	To develop a three-gene multilocus sequence typing (MLST) scheme for TPE, and find markers that may be useful for molecular distinction of TPE strains	A total of twenty-two complete strain types were identified; two strain types in clinical samples from Vanuatu (5q11/ak and 5q12/ak), nine strain types in clinical samples from Ghana (3q12/ah, 4r12/ah, 4q10/j, 4q11/ah, 4q12/ah, 4q12/v, 4q13/ah, 6q10/aj, and 9q10/ai), and twelve strain types in laboratory strains and published genomes (2q11/ae, 3r12/ad, 4q11/ad, 4q12/ad, 4q12/ag, 4q12/v, 5r12/ad, 6r12/x, 6q11/af, 10q9/r, 10q12/r, and 12r12/w).

## 4. Discussion

A limited number of studies in the endemic countries show that yaws eradication in Africa needs more attention. Our review highlights a prevalence ranging from 0.50% to 43.0% in 11 African countries. Underreporting and underestimation result in data limitations, as the actual cases are predominant in hard-to-reach rural areas with limited or no access to health facilities. Secondly, this could be because of the need for more training of health personnel to identify and report cases. The weakness of the data arising from the limitations of the studies selected for review suggests a need for more research into yaws endemicity in the African region.

Yaws can be diagnosed when patients present with clinical signs and symptoms associated with the disease, and it is often augmented with serological testing, which employs the use of combined treponemal assay, Treponema pallidum particle agglutination assay (TPPA) and a non-treponemal assay, rapid plasma regain assay (RPR) [42]. While clinical and serological diagnosis of yaws has brought much progress towards eradication, these methods have yet to be definitive and must be supported by laboratory diagnostics [35,38,43]. Fortunately, newer point-of-care treponemal and non-treponemal assays have increasingly taken on the role of traditional lab-based assays [14,42,44]. However, although treponemal tests are more specific, they remain positive for life [37]. Recently, diagnosis by clinical examination may be challenging because the lesions can resemble several other tropical skin diseases [45]. For example, ulcerative skin lesions like that caused by *Haemophilus ducreyi* to that seen in early yaws lesions [46,47] complicate diagnosis as serological testing does not provide a differential diagnosis [42]. Patients who were serologically diagnosed negative for yaws were diagnosed positive using PCR [44]. Again, molecular methods have been employed to differentiate between latent and active yaws. In some cases, patients diagnosed with active yaws have been molecularly diagnosed as latent, and those thought to be yaws were caused by different organisms [35,44]. In this review, only two studies combined clinical, serological, and molecular detection by PCR [24,25]. Hence, countries that have employed clinical and serological diagnosis over the years can improve surveillance by using PCR and mapping to track progress towards eradication.

Penicillin-based treatment (PBT) was the earliest treatment regimen that the WHO recommended [8,48] and was used in many eradication campaigns, achieving up to 98% reduction in global yaws prevalence [8,9,44]. Despite the success brought by the PBT, logistical challenges such as a secured cold chain and accessories and trained health professionals to administer injections created a barrier to eradication efforts in yaws endemic settings [3,15,16] led to the introduction of oral azithromycin, following studies conducted in Papua New Guinea [11] and in Ghana [12]. These studies showed that a single dose of azithromycin at 30 mg/kg had the same efficacy as a single dose of injectable benzathine penicillin, supporting WHO policy for the use of azithromycin for yaws eradication in resource-constrained communities [37]. Hence, the WHO suggested the use of the “Morges strategy” for yaws eradication that employs the use of azithromycin for mass drug administration (MDA) in two different ways: an initial round of total community treatment (TCT) and a subsequent MDA or to switch to a strategy of treating active cases and their contacts (total targeted treatment, TTT) [13]. Using the approach in Papua New Guinea’s endemic island has shown significant progress; however, individuals absent during mass treatment had yaws reactivated [49]. This review revealed that adopting the recommended approach, especially in endemic countries in western Africa, is yet to be considered. To achieve the complete interruption of yaws transmission, stakeholders must support endemic countries to implement the approach for achieving a successful TCT developed and employed in the Congo Basin [40].

The limited reliability of clinical diagnosis of yaws and the recognition that other organisms can cause clinically similar skin lesions in yaws-endemic countries [46,47] cause problems in clinical case reporting. It is essential to quickly strategize to integrate yaws into a comprehensive diagnostic scheme, such as point-of-care serological tests and new molecular tests [50]. Another challenge to disrupting yaws transmission is the need for epidemiological data [15]. Currently, there are nine African countries: Benin, Cameroon, Central African Republic, Côte d’Ivoire, Ghana, Liberia, Congo, Democratic Republic Congo and Togo known to be endemic [51]. Improving this situation will require a significant increase in the scale and speed of mapping to strengthen epidemiological data and inform decisions about the interventions and resources necessary for a successful eradication effort to yield the needed benefit [15]. Again, recently, treatment failure has been reported in the Pacific region, where the “Morges strategy” was employed [49], and the report showed macrolide resistance mediated by point mutations in the 23S rRNA gene [52,53,54], which was associated with prior exposure to other macrolides [49,55]. Further studies should be conducted to evaluate the current state of macrolide resistance, and efforts should be made to combat this emerging resistance. Regular surveillance to monitor the effectiveness of the treatment strategies in endemic countries will be crucial for yaws elimination.

## 5. Conclusions

The recommended strategies and diagnostic tools adopted by the WHO show promising guidelines for eradicating yaws. With government sponsorship and support, previously endemic countries like India and Ecuador have successfully eliminated yaws. The remaining endemic countries may capitalize on the simple, cheap, and well-tolerated oral treatment that has proven effective, point-of-care diagnostic tests that can enable an initial identification of potential yaws cases and new molecular tests that have become available. These endemic countries should harness the increased interest and support within the academia and global health communities for yaws eradication efforts and increase governmental support for greater research output related to yaws. In addition, control programs in endemic countries should increase advocacy to transmit to the broader public health community the need to eliminate such neglected tropical diseases. These programs can benefit from the knowledge and experience of past yaws campaigns and other international NTD control initiatives. There is still an excellent prospect for eradication if countries embark on integrated disease control efforts to increase sustainability and improve the quality of life for people living with this NTD in poor communities.

## 6. Limitations

This study was limited to Africa and did not provide a global perspective. Additionally, the data used in the review did not include data on specific countries’ national control programs.

## Data Availability

Data will be made available upon request.
